# Refining the Pulmonary and Functional Competencies in a Male Patient With Guillain-Barré Syndrome

**DOI:** 10.7759/cureus.45101

**Published:** 2023-09-12

**Authors:** Sawari S Bhagwatkar, Pallavi Harjpal

**Affiliations:** 1 Physiotherapy, Ravi Nair Physiotherapy College, Datta Meghe Institute of Higher Education and Research, Wardha, IND; 2 Neurophysiotherapy, Ravi Nair Physiotherapy College, Datta Meghe Institute of Higher Education and Research, Wardha, IND

**Keywords:** physical therapy, case report, rehabilitation, physiotherapy, guillain-barré syndrome

## Abstract

Guillain-Barré syndrome (GBS) is the most prevalent form of autoimmune-related acute demyelinating polyneuropathy that affects people of any age group. Its global prevalence is 1.9 per 100,000 people. Acute or subacute symmetrical motor and sensory neuropathy involving several peripheral nerves is referred to as GBS. It typically occurs after an infection caused by a virus, but infrequently with surgery or vaccination. There are different variants of GBS, like acute sensory axonal neuropathy, acute motor axonal neuropathy, and Miller-Fisher syndrome. Motor paralysis that affects distal muscles more than proximal muscles and is more pronounced and symmetrical may be a presenting symptom of GBS. Over the course of several days, it starts in the legs and progresses to the arms, face, and eyes. Reflexes may be missing, bifacial weakness may be present, severe cases result in respiratory paralysis, and autonomic abnormalities may be rare. Patients with GBS exhibit anti-ganglioside antibodies that seem to react with antigens found in some previous infectious pathogens' lipopolysaccharides. These antibodies target gangliosides, like GM1, which are dispersed within the myelin of the peripheral nervous system. There are three phases: acute, plateau, and recovery. Only plasmapheresis and intravenous immunoglobulin have shown effective recovery.

A 24-year-old male presented with weakness of the bilateral lower limb associated with fever and breathlessness. The range of motion of hip flexion was reduced to 45 degrees, and muscle power was also reduced. For hip flexors, it was 3/5; for knee flexors and extensors, it was 4/5; and for ankle plantar flexors and dorsiflexors, it was 2/5. Investigations like a complete blood count (CBC), cerebrospinal fluid (CSF) examination, and nerve conduction velocity (NCV) were done. Post-diagnosis, the patient received an intravenous immunoglobulin (IVIG) dose; the same was managed by neurophysiotherapy, and after treatment, the patient was functionally independent. According to the findings of our study, neurorehabilitation resulted in favorable outcomes, shortened the length of the hospital stay, and enabled him to return to his desk job.

## Introduction

Guillain-Barré syndrome (GBS) is the most prevalent and severe form of acute paralytic neuropathy which affects over 100,000 people worldwide each year [1]. Muscular hypotonia, the fast onset of generalized muscle weakness (the critical course), and profound or absent hypoactive reflexes are the hallmarks of acute flaccid paralysis (AFP). There are multiple recognizable subtypes of Guillain-Barré syndrome, each with unique clinical and pathological characteristics. Twenty to thirty percent of cases with Guillain-Barré syndrome have a severe, generalized presentation of respiratory failure [1]. An acute inflammatory immune-mediated polyradiculoneuropathy, GBS usually manifests as tingling, a weakening of the muscles, and discomfort [2]. The diagnosis criteria for GBS are progressive, relatively symmetrical weakness in more than one limb, decreased or absent myotatic reflexes (areflexia), or distal areflexia with proximal hyporeflexia, along with albumin cytologic dissociation. Guillain-Barré syndrome is an inflammatory polyneuropathy marked by a quick progression, precarious ambulation, symmetric muscular weakness, and hypo- or areflexia. At least initially, the weakening is typically primarily distal, and many patients experience neuropathic discomfort. Ascending paralysis is how GBS typically manifests [3]. Guillain-Barré syndrome, an uncommon but potentially fatal immune-mediated illness of the peripheral nerves and nerve roots, is frequently brought on by infections. Because of this, the prevalence of GBS may increase during infectious disease outbreaks, as it did with the Zika virus outbreaks in French Polynesia in 2013 and in South America in 2015 [4]. Due to its growing geographic reach, Zika fever is now recognized as an emerging disease of arboviral origin.

A degenerative nerve complication, GBS, may develop following the resolution of a fever as well as other related symptoms brought on by one or more neurotropic viruses [5]. It is now understood that there are various types of GBS, such as acute inflammatory demyelinating polyneuropathy, in which the immune system's damage affects the myelin sheath and its companion Schwann cells, and acute motor axonal neuropathy, in which the nerve axon membranes serve as the main focus. Earlier, it was believed that the severity of GBS was correlated with the degree of axonal damage [3]. Plasma exchange (200-250 ml plasma/kg of body mass over five sessions) and intravenous immunoglobulin (IVIG) (0.4 g/kg of body mass each day for five days) are two treatments for GBS. Immunoglobulin is frequently more accessible than plasma exchange and is typically the chosen course of treatment since it is easier to give. Intravenous immunoglobulin and plasma exchange are the only treatments for GBS that have been demonstrated to be effective. Despite the fact that corticosteroids might be useful for reducing inflammation [4], prior research has shown the value of neurorehabilitation and cardio-respiratory exercise, including active-assisted training and progressing to strengthening exercises [6], along with physical rehabilitation and routine vitals checks. Functional independence was achieved by the patient in the absence of lingering frailty within a month. Early physical care is essential to aiding such individuals in regaining their functional independence [7].

## Case presentation

Patient information

An outpatient clinic recommended a 24-year-old male patient to our hospital. His presenting symptoms were weakness in bilateral lower limbs, which was gradual in onset and was associated with a high-grade fever and breathlessness. The patient visited a local hospital, where he was admitted for three days. The patient was treated with some antibiotics and 0.9% normal saline solution (NaCl), but no symptomatic relief was experienced by the patient. He was referred to our hospital, where he underwent some investigations like a cerebrospinal fluid (CSF) examination, which revealed elevated protein levels, i.e., albumin-cytological dissociation, and a nerve conduction velocity test that indicated F-wave delay in the tibial and peroneal nerves. The patient was in the ICU for three days, maintaining oxygen saturation in room air, followed by a stay of six days in the neurology ward, where he was provided with an IVIG dose for six days, followed by physiotherapy.

Clinical findings

The patient had a mesomorphic build and had a keen awareness of place, time, and people. Both his upper and lower limbs had all of their sensory abilities. As there was bilateral lower limb weakness, muscle power was reduced in both lower extremities. By using the Medical Research Council grading, hip flexors were 3/5, 4/5 in knee flexors and extensors, and 2/5 in ankle plantar flexors and dorsiflexors. There is no involvement of the upper limbs. The range of motion of hip flexion was reduced to 45 degrees due to reduced muscle power. On assessing reflexes, the biceps, triceps, and supinator reflexes were intact, but knee jerks were diminished, and the planter was the flexor. Proprioception was affected in the lower extremities at distal joints. While assessing muscle tone by the modified Ashworth scale, tone for bilateral upper and lower extremities was normal. On auscultation, there was reduced air entry in the bilateral lower zones. There was no bowel or bladder involvement, but respiratory complications were present with a Hughes severity score of 4/6.

Clinical diagnosis

**Figure 1:**
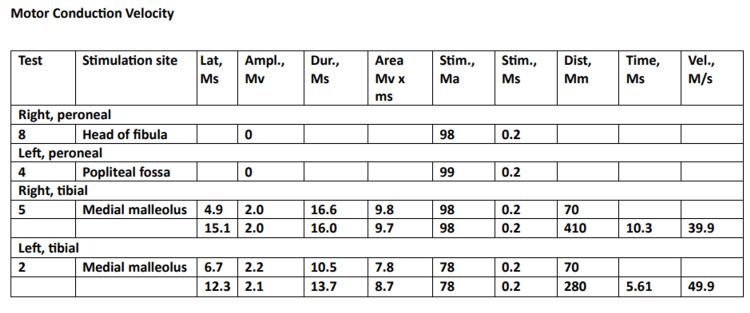
Nerve conduction velocity reports of the patient Lat: latency; ms: milliseconds; Ampl: amplitude; mV: millivolts; Dur: duration; ms: milliseconds; mV X ms: millivolts X milliseconds; Stim: stimulation; ma: milliamperes; mm: millimeters; Vel: velocity; m/s: meter/seconds

Based on the clinical findings, a lumbar puncture was performed for CSF analysis, which revealed an increased protein count of 55 mg/dL and a cell count of 15-20 cells per μL. Nerve conduction velocity revealed compound muscle action potential (CMAP) amplitude could not be elicited in the bilateral peroneal nerve. The F-wave latency could not be elicited in bilateral tibial nerves, as indicated in Figure 1.

He was diagnosed with a case of sensory-motor polyneuropathy. As the patient was in the ICU for three days, there were no signs of hospital-acquired neuromuscular disorders.

Physiotherapy interventions

The patient was taught range-of-motion exercises and the importance of routinely switching positions in order to maintain joint mobility and integrity. Table 1 provides the therapy protocol.

**Table 1:** Physiotherapy interventions for the patient ROM: range of motion; ADLs: activities of daily living

Problem identified	Cause of the problem	Goal framed	Physiotherapy intervention	Rationale
Reduced air entry into the lungs	Diaphragm and intercostal muscular weakness	Within two weeks, the patient can carry out all the moderately demanding exercises in the absence of fatigue.	Exercises like pursed lip breathing, thoracic expansion, and incentive spirometry, as well as diaphragmatic breathing (Figure 2)	Diaphragmatic breathing activates the diaphragm, which is the primary muscle used for respiration.
Accumulation of secretions leading to increased work of breathing.	It may be due to aspiration and pneumonia, which are respiratory complications.	Within two weeks, the patient’s secretions will be reduced and their breathing will be normalized.	Postural drainage against gravity positions can be given, focusing on zones where secretions are present. An active breathing technique and autogenic drainage were provided.	Secretions are removed, and the airway is cleared.
Decreased range of motion.	Due to muscle weakness	Within two weeks, the patient will be able to engage in all activities without any difficulties.	Active-assisted workouts are followed by active range of motion exercises for bilateral lower limbs and stretching (Figure 3).	Active ROM exercises and stretching will activate the muscle spindle and agonist muscles, which will improve ROM and joint integrity.
Weakness of muscles of the lower extremity	Decreased nerve conduction and weakness brought on by the illness and hospitalization	Within two weeks of the intervention, the patient's limbs will eventually recover their previous strength.	Strengthening exercises for the lower limbs using a weight cuff (starting at 1/2 kg and working up to 1 kg). strengthening of the hips and quadriceps together	It will improve muscle strength and muscle performance.
Postural deviation	Due to prolonged hospitalization and being bedridden	Postural deviation will be brought back to normal at the end of two weeks.	Postural corrective exercise, positioning, and chest binders	Normal posture will be regained by the patient.
Proprioception impairment	Bedridden for a long period of time	With the right training, proprioception will return in three weeks.	Joint compression and training with a proprioceptive trainer	Proprioception is regained to normal.
Abnormal gait	Prolonged hospitalization, affection for proprioception	The patient will be able to move around easily with a healthy walking stride after three to four weeks of gait training.	Exercises that strengthen the quadriceps include single-leg stance, ankle dorsiflexion, toe and heel raises, side leg raises, gait training, sitting marching, squatting, and knee-to-chest.	Improved gait pattern
Reduced ADL’s	Muscle performance is reduced.	After receiving treatment for five to six weeks, the patient can return to work.	Promote utilizing your extremities for ADLs.	It will make the patient functionally independent.

**Figure 2:**
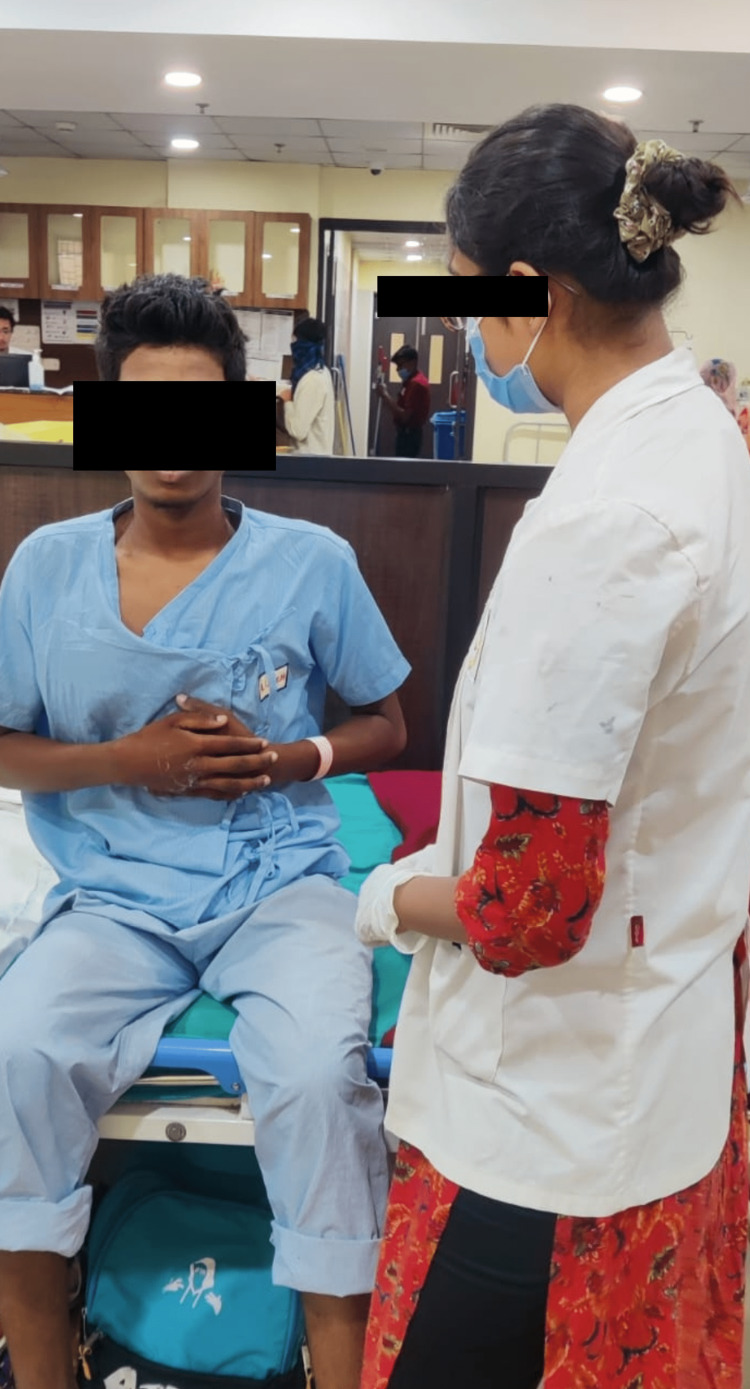
Patient performing deep breathing exercises

**Figure 3:**
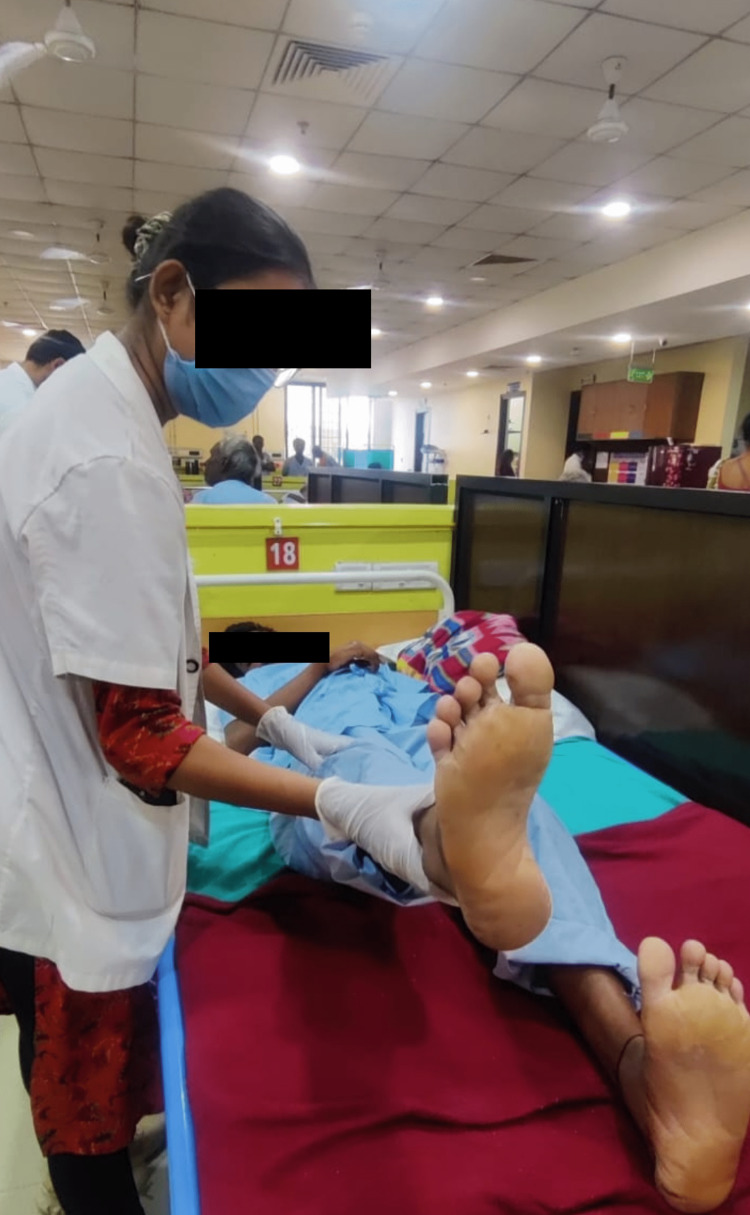
Active-assisted exercise for hip flexion

Figure 2 and Figure 3 are part of the intervention protocol.

The patient received this regimen once a day, performing each exercise 10 times with appropriate breaks in between. The patient was instructed to engage in breathing exercises and active limb movement throughout the day in addition to this course of treatment.

Follow-up and outcome measures

There was an improved range of motion for bilateral lower limbs, and muscle power was increased to 4/5 on the Medical Research Council Scale for both lower extremities. Muscle tone was brought back to normal post-rehabilitation. The patient was able to regain all normal functional activities after rehabilitation. The functional independence measure (FIM) score increased from 80/126 to 120/126. After therapy, the Hughes severity score went from 4/6 to 0/6. On the Berg balance scale, the score increased to 48/56.

Results

Activities of daily living (ADL) routine and formerly difficult movement transfers (squatting and stair-climbing sit-to-stand) were made possible for the patient. By the time of discharge, the Hughes severity scale had returned to its normal value of 0/6. After rehabilitation, the patient was functionally independent. The power of hip flexors, knee flexors, extensors, and ankle plantar flexors was recovered. The range of motion of hip flexion was improved to 110 degrees. The patient's quality of life was improved by all of this, and he was able to get back to his desk job quickly.

## Discussion

In this case report, we are discussing a case with Guillain-Barré syndrome, which is an autoimmune inflammatory demyelinating disease of peripheral nerves, and the effects of early physiotherapy rehabilitation on improving functional independence in patients and preventing further complications. The therapy of GBS has evolved over the past 10 years from basic supportive care and complication management to an active approach (plasma exchange and intravenous immunoglobulin infusion) that reduces the severity of the disease, particularly in patients who are severely affected [8]. The patient in this case was also provided with IVIG treatment. In this case, treatment of the underlying cause, the long-term recovery of the patient, and the acute care of paralysis and its complications were the main focuses of our management, which was in accordance with earlier studies [9-11].

According to a study that shows that rehabilitation has a significant role in accelerating recovery, the recovery in ADLs was only extremely mild [9]. Studies have shown that early physical therapy helps severely ill ICU patients recover quickly [10-12]. Our key areas of attention were weakness and tiredness, and the results improved in line with earlier research showing that emphasizing fatigue produces better immediate outcomes. Studies on early-focused physiotherapy, which encourages an early return to activities like sports, have also been published [13].

## Conclusions

According to the findings of our study, neurophysiotherapy rehabilitation results in favorable outcomes, shortens the length of the patients' hospital stays, and enables them to return to their jobs. Further evidence from the trial supports the proposed physiotherapy protocol's value in treating acute GBS cases. The functional recovery of a patient with GBS depends on various factors, such as the severity of the condition, nerve involvement, early diagnosis, timely administration of pharmacotherapy, and a comprehensive therapy program. These factors can help prevent prolonged bed rest complications and expedite functional recovery. It also concludes that starting an early rehabilitation program plays a very important role and can be very effective in GBS patients, as it will make them functionally independent and also improve their quality of life.
